# THE USE OF MICRO-CREDENTIALS IN OTHER DISCIPLINES: EXAMPLES FROM THE FIELD

**DOI:** 10.1093/geroni/igae098.1002

**Published:** 2024-12-31

**Authors:** Bronwyn Keefe

**Affiliations:** Boston University, Boston, Massachusetts, United States

## Abstract

This presentation will describe training partnerships between Boston University’s Center for Aging & Disability Education & Research (CADER) and state and community-based agencies that provide health and social services to older adults. The workforce is looking for micro-credentials to build their competencies just as state and local agencies seek partnerships with academic institutions to build career ladders and finetune skills for their employees. Skills-based hiring and employers’ willingness to move beyond requiring college degrees has grown significantly, driven by the tight job market and employer concerns about diversity and equity. Many practitioners in the field of aging have not received any formal education in working with older adults, while those who seek services are more diverse than ever, and they have a complexity of medical and social concerns. We need a trained and competent workforce to provide high-quality care for older adults. It is imperative to build reciprocal academic and community-based partnerships to further prepare and retool the aging workforce, especially during a great exodus of social service practitioners. Training through CADER’s online certificates has led to statistically significant increases in core competencies in aging and increased knowledge and confidence. Almost half (47%) have taken CADER training for career advancement and personal interest, while the remaining (53%) participated because of work-mandated training initiatives, and a quarter (25%) stated that they had received a pay raise, title change, or promotion since taking the training.

